# Design of Nanoscaled Surface Morphology of TiO_2_–Ag_2_O Composite Nanorods through Sputtering Decoration Process and Their Low-Concentration NO_2_ Gas-Sensing Behaviors

**DOI:** 10.3390/nano9081150

**Published:** 2019-08-11

**Authors:** Yuan-Chang Liang, Yen-Chen Liu

**Affiliations:** Department of Optoelectronics and Materials Technology, National Taiwan Ocean University, Keelung 20224, Taiwan

**Keywords:** sputtering, surface decoration, nanostructured surface, composite nanorods

## Abstract

TiO_2_–Ag_2_O composite nanorods with various Ag_2_O configurations were synthesized by a two-step process, in which the core TiO_2_ nanorods were prepared by the hydrothermal method and subsequently the Ag_2_O crystals were deposited by sputtering deposition. Two types of the TiO_2_–Ag_2_O composite nanorods were fabricated; specifically, discrete Ag_2_O particle-decorated TiO_2_ composite nanorods and layered Ag_2_O-encapsulated TiO_2_ core–shell nanorods were designed by controlling the sputtering duration of the Ag_2_O. The structural analysis revealed that the TiO_2_–Ag_2_O composite nanorods have high crystallinity. Moreover, precise control of the Ag_2_O sputtering duration realized the dispersive decoration of the Ag_2_O particles on the surfaces of the TiO_2_ nanorods. By contrast, aggregation of the massive Ag_2_O particles occurred with a prolonged Ag_2_O sputtering duration; this engendered a layered coverage of the Ag_2_O clusters on the surfaces of the TiO_2_ nanorods. The TiO_2_–Ag_2_O composite nanorods with different Ag_2_O coverage morphologies were used as chemoresistive sensors for the detection of trace amounts of NO_2_ gas. The NO_2_ gas-sensing performances of various TiO_2_–Ag_2_O composite nanorods were compared with that of pristine TiO_2_ nanorods. The underlying mechanisms for the enhanced sensing performance were also discussed.

## 1. Introduction

The development of chemosensors made from semiconductor oxides has recently become a key research topic [1,2]. Therefore, the development of highly responsive sensing oxide devices toward specific harmful gases has attracted interest in industry. For the gas sensor applications, one-dimensional (1D) metal oxides usually show better performance in comparison with their thin-film or bulk counterparts because of their high surface-to-volume ratio [3,4,5,6]. In particular, gas sensors based on 1D titanium dioxide (TiO_2_) nanostructures have received considerable attention because they can be fabricated with diverse chemical and physical methods; moreover, TiO_2_ has been shown to be favorable for the detection of diverse harmful gases and volatile organic vapors at elevated temperatures [5,7,8].

Recently, combining n-type oxides with p-type semiconductor oxides to form a heterogeneous structure has attracted great attention due to this combination’s enhanced gas-sensing performance toward target gases [9,10,11]. The existence of the interfacial potential barrier at the p–n junctions of the heterogeneous structure play an important role in improving the gas-sensing performance of the constituent oxides. Several p–n junction-based sensors made from different material systems and configurations have been proposed. For examples, Woo et al. reported a discrete configuration of p-type Cr_2_O_3_ nanoparticles on the surfaces of ZnO nanowires; this p–n heterostructure enhances gas selectivity and sensitivity toward trimethylamine [11]. The decoration of NiO nanoparticles in porous SnO_2_ nanorods remarkably enhances the gas-sensing response to ethanol as compared with pristine SnO_2_, which can be attributed to the formation of NiO–SnO_2_ p–n heterojunctions [12]. P-type Ag_2_O phase-functionalized In_2_O_3_ nanowires shows an improved gas-sensing performance toward NO_2_ gas [13]. However, reports on the incorporation of p-type oxides into n-type TiO_2_ nanostructures to form a p–n junction gas sensor are limited in number. 

In this study, 1D TiO_2_–Ag_2_O p–n heterogeneous structures are synthesized through the combination of hydrothermal growth and sputtering methods. Ag_2_O is a p-type semiconductor oxide; it has previously been used as a gas-sensing material [13]. Moreover, Ag_2_O crystals with various morphologies can be synthesized through chemical or physical routes [14,15,16]. Notably, Ag_2_O particles are mostly synthesized through chemical routes but, using such chemical routes, it is hard to control the decoration morphology of the Ag_2_O crystals on 1D nanostructures [15]. By contrast, the fabrication of Ag_2_O through a physical method (sputtering) is advantageous concerning the control of the Ag_2_O content, crystalline quality, and coverage morphology on 1D TiO_2_. In this study, two types of TiO_2_–Ag_2_O composite nanorods are synthesized. By controlling the sputtering duration of the Ag_2_O, discrete Ag_2_O particle-decorated TiO_2_ nanorods and Ag_2_O layers encapsulating TiO_2_ nanorods are fabricated. The Ag_2_O coverage morphology effects on the low-concentration NO_2_ gas-sensing performance of the TiO_2_–Ag_2_O p–n composite nanorods are systematically investigated in this study.

## 2. Materials and Methods 

**Figure 1:**
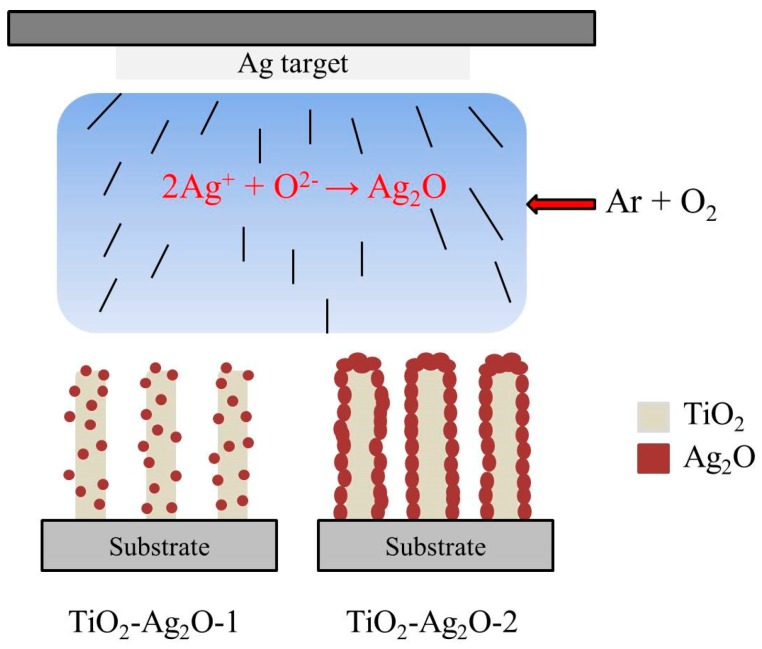
Sample configurations of the TiO_2_–Ag_2_O-1 and TiO_2_–Ag_2_O-2 composite nanorods synthesized with various sputtering durations of Ag_2_O.

In this study, TiO_2_ nanorods were grown on fluorine-doped SnO_2_ (FTO) glass substrates. First, 0.25 mL of TiCl_4_ and 19 mL HCl were added to 11 mL deionized water and then stirred to obtain a transparent solution for the hydrothermal growth of TiO_2_ nanorods. The hydrothermal reaction was conducted at 180 °C for 3 h. For the preparation of TiO_2_–Ag_2_O composite nanorods, Ag_2_O crystals were decorated onto the surfaces of the TiO_2_ nanorod template via sputtering. Radiofrequency magnetron sputtering of Ag_2_O was conducted using a silver metallic target in an Ar/O_2_ (Ar:O_2_ = 5:2) mixed ambient. The sputtering deposition temperature of the Ag_2_O was maintained at 200 °C. The gas pressure during sputtering deposition was fixed at 20 mTorr and the sputtering power was fixed at 50 W for the silver target. Two sets of TiO_2_–Ag_2_O composite nanorods with Ag_2_O sputtering durations of 130 s and 270 s were prepared; these were represented as TiO_2_–Ag_2_O-1 and TiO_2_–Ag_2_O-2, respectively, in this study. The sample configurations of the TiO_2_–Ag_2_O-1 and TiO_2_–Ag_2_O-2 composite nanorods are shown in Figure 1.

An X-ray diffractometer (XRD; D2 PHASER, Bruker, Karlsruhe, Germany) was used to analyze the crystal structures of the TiO_2_–Ag_2_O composite nanorods. Scanning electron microscopy (SEM; S-4800, Hitachi, Tokyo, Japan) and transmission electron microscopy (HRTEM; JEM-2100F, JEOL Tokyo, Japan) were used to characterize the morphology and detailed microstructures of the composite nanorod samples. The attached energy dispersive X-ray spectroscopy (EDS) of TEM was used to investigate the composition and composition distribution of the nanorod samples. Moreover, X-ray photoelectron spectroscopy (XPS; ULVAC-PHI XPS, ULVAC, Chigasaki, Japan) was used to characterize the elemental binding states of the synthesized samples. 

The response of pure TiO_2_ nanorods and the TiO_2_–Ag_2_O nanocomposites to NO_2_ was tested in a vacuum test chamber. Silver electrodes were laid on the surfaces of the samples to form electric contacts for measurements. An Agilent B2911A meter measured the resistance variation of the nanorod sensors at a constant potential of 5 V as a function of time. Constant dry air was used as the carrier gas and the desired concentration of NO_2_ gas (0.5, 1.5, 3.0 ppm) was introduced into the test chamber. A direct heating approach was used to operate the sensors at elevated temperatures in the range of 200–300 °C. 

## 3. Results and Discussion

**Figure 2:**
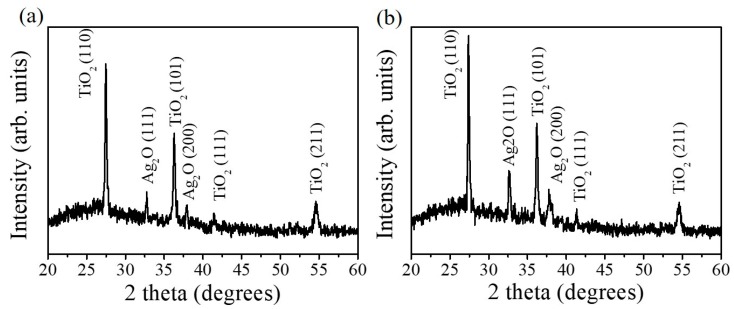
XRD patterns of various composite nanorods: (**a**) TiO_2_–Ag_2_O-1, (**b**) TiO_2_–Ag_2_O-2.

X-ray diffractometer (XRD) patterns of TiO_2_–Ag_2_O composite nanorods with various Ag_2_O thin-film sputtering durations are shown in Figure 2. The distinct Bragg reflections centered at 27.46°, 36.05°, 41.22°, and 54.33° correspond to the crystallographic planes (110), (101), (111), and (211) of the rutile TiO_2_ phase, respectively (JCPDS no. 00-004-0551). Moreover, the Bragg reflections centered at 32.72 ° and 37.98 ° are assigned to the crystallographic planes of cubic Ag_2_O (111) and (200), respectively (JCPDS no. 00-012-0793). The XRD results reveal that highly crystalline rutile TiO_2_-cubic Ag_2_O composite nanorods were formed through the sputtering deposition of Ag_2_O thin films onto the surfaces of the TiO_2_ nanorods, and no other impurity peak was observed. As expected, the intensity of the Ag_2_O Bragg reflections peaks increased with the increase of the Ag_2_O thin-film sputtering duration, revealing an increased Ag_2_O phase content in the composite nanorods.

**Figure 3:**
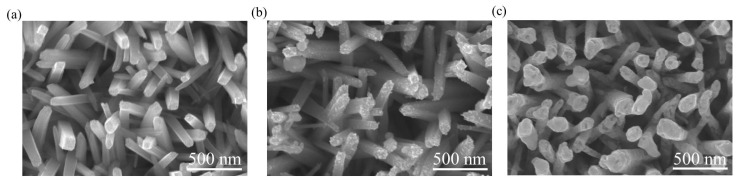
SEM images of various nanorods: (**a**) TiO_2_, (**b**) TiO_2_–Ag_2_O-1, (**c**) TiO_2_–Ag_2_O-2.

Figure 3a shows the scanning electron microscopy (SEM) image of the as-synthesized TiO_2_ nanorods. The TiO_2_ nanorods had a rectangular cross-section crystal feature with an average diameter of approximately 100 nm; the side facets of the nanorods were smooth. Figure 3b presents the SEM image of the TiO_2_–Ag_2_O-1 composite nanorods. The surface morphology of the composite nanorods reveals that a small amount of nanoparticle-like crystals was decorated onto the surfaces of the TiO_2_ nanorods. The nanoparticle-like crystals dispersed separately on the surfaces of the TiO_2_ nanorods. Figure 3c shows the SEM image of the TiO_2_–Ag_2_O-2 composite nanorods. The TiO_2_ nanorods were homogeneously encapsulated by the aggregation of massive Ag_2_O nanoparticles, resulting in the irregular-shaped cross-section crystal feature of the composite nanorods. Detailed TEM analyses were performed to further confirm the morphology change of the TiO_2_–Ag_2_O composite nanorods prepared at various Ag_2_O sputtering durations.

**Figure 4:**
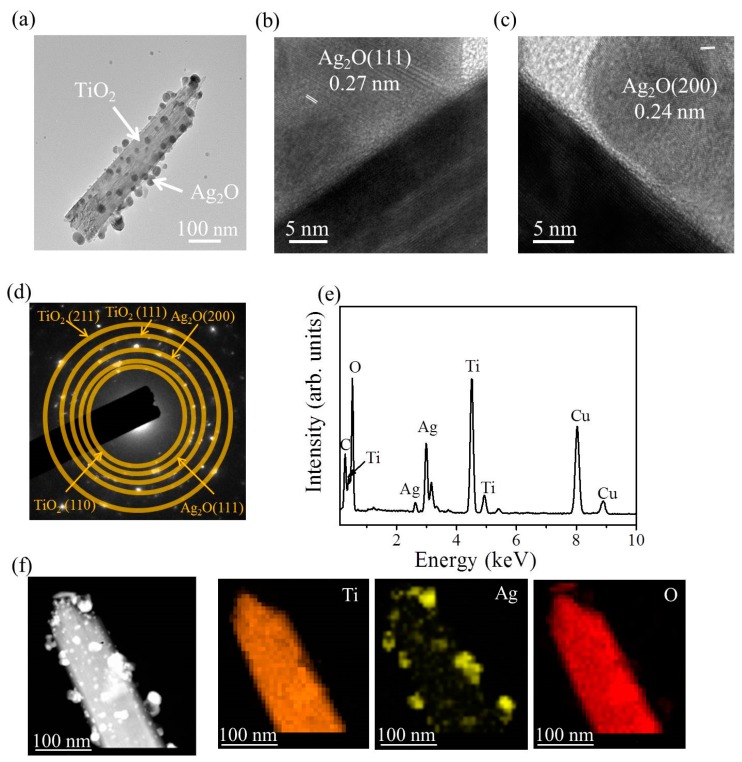
TEM analysis of the TiO_2_–Ag_2_O-1 composite nanorods: (**a**) Low-magnification TEM image of the TiO_2_–Ag_2_O-1 composite nanorod. (**b**,**c**) High-resolution TEM images taken from various regions of the composite nanorod. (**d**) Selected area electron diffraction (SAED) pattern of several TiO_2_–Ag_2_O-1 composite nanorods. (**e**) Energy dispersive X-ray spectroscopy (EDS) spectrum of the composite nanorod. (**f**) Ti, Ag, and O elemental mapping images taken from the selected composite nanorod.

Figure 4a shows the low-magnification transmission electron microscopy (TEM) image of a TiO_2_–Ag_2_O-1 composite nanorod. A small amount of Ag_2_O particles was dispersedly decorated on the surface of the TiO_2_ nanorod via the sputtering growth of the Ag_2_O. The high-resolution TEM images shown in Figure 4b,c indicate distinct lattice fringes in the Ag_2_O particle. Moreover, the lattice fringe distance of approximately 0.24 nm was assigned to the crystallographic plane spacing of cubic Ag_2_O (200). Figure 4d exhibits the selected area electron diffraction (SAED) pattern of several TiO_2_–Ag_2_O-1 composite nanorods. Several clear diffraction rings associated with (111) and (200) planes of the Ag_2_O and (110), (111), and (211) planes of the rutile TiO_2_ were observed in the SAED pattern. This demonstrates the good crystallinity of the composite nanorods and indicates that these composite nanorods have a polycrystalline nature. Figure 4e presents the EDS spectrum of a TiO_2_–Ag_2_O-1 nanorod. In addition to carbon and copper signals originating from the TEM grid, Ti, Ag, and O elements were detected in the selected heterostructure and no other impurity atom was detected. The EDS elemental mapping images taken from the TiO_2_–Ag_2_O-1 nanorod are presented in Figure 4f. The Ti signals were homogeneously distributed over the region of the nanorod template. By contrast, the Ag signals were mainly distributed on the outer region of the composite nanorod; the distribution of Ag signals was discrete and randomly decorated on the TiO_2_ surface. A good Ag_2_O particle-decorated TiO_2_ nanorod with a dispersive particle decoration feature was obtained in the TiO_2_–Ag_2_O-1 composite nanorods.

**Figure 5:**
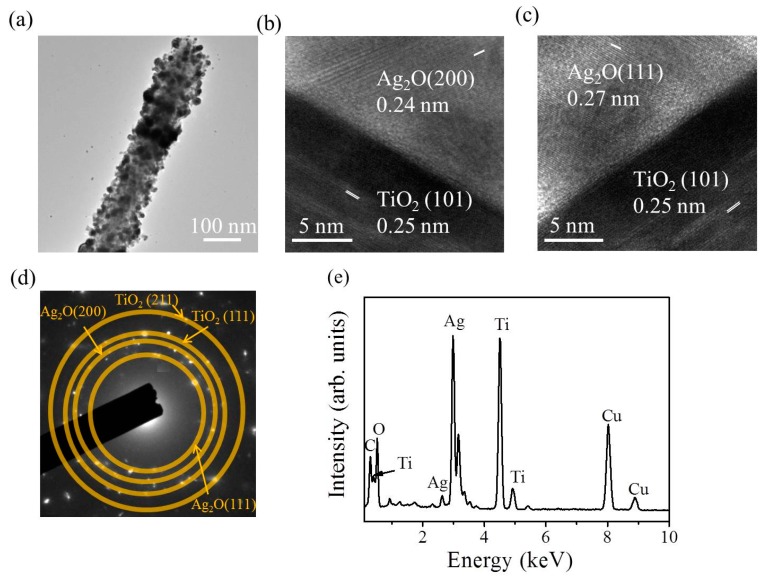
TEM analysis of the TiO_2_–Ag_2_O-2 composite nanorods: (**a**) Low-magnification TEM image of the TiO_2_–Ag_2_O-2 composite nanorod. (**b**,**c**) High-resolution TEM images taken from various regions of the composite nanorod. (**d**) SAED pattern of several TiO_2_–Ag_2_O-2 composite nanorods. (**e**) EDS spectrum of the composite nanorod.

Figure 5a shows the low-magnification TEM image of the TiO_2_–Ag_2_O-2 composite nanorod. In comparison with Figure 4a, the distribution density of the Ag_2_O particles on the surface of the TiO_2_ nanorod was denser and many particles were clustered, resulting in a rugged surface feature of the composite nanorod. The tiny Ag_2_O particles aggregated together and fully encapsulated the surface of the TiO_2_ nanorod. A clear heterointerface was observed between the TiO_2_ and Ag_2_O (Figure 5b,c). The distinct lattice fringes in the inner and outer regions of the composite nanorod in Figure 5b,c demonstrated a good crystallinity of the composite nanorod. The SAED pattern in Figure 5d supports the good crystallinity of the composite nanorods, as revealed in the high-resolution TEM images of the selected composite nanorod, and is also in agreement with the XRD result. Figure 5e shows the corresponding EDS spectrum of the TiO_2_–Ag_2_O-2 composite nanorod. Besides the carbon and copper signals and the elements of Ag, Ti, and O, no other impurity atom was detected from the selected composite nanorod. Notably, the relative intensity of the Ag signal was more intense than that of the TiO_2_–Ag_2_O-1 nanorod in Figure 4e, revealing a higher Ag content in TiO_2_–Ag_2_O-2 due to the prolonged sputtering duration of Ag_2_O.

**Figure 6:**
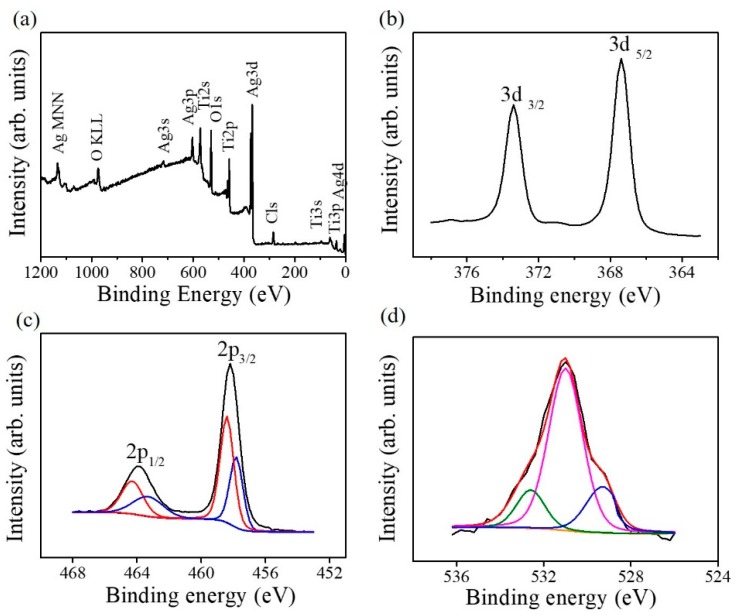
XPS analysis of the TiO_2_–Ag_2_O-1 composite nanorods: (**a**) Survey scan spectrum. (**b**) Ag 3d narrow scan spectrum. (**c**) Ti 2p narrow scan spectrum. The red curve is associated with the contribution of the Ti^4+^ valance state and the blue curve originated from the Ti^3+^ valence state. (**d**) O1s narrow scan spectrum. The blue and pink curves are ascribed to the lattice oxygen in Ag_2_O and TiO_2_, respectively. Moreover, the green curve is ascribed to external absorbed −OH groups or water molecules on the surfaces of the composite nanorods.

Figure 6a shows the X-ray photoelectron spectroscopy (XPS) survey scan spectrum of the TiO_2_–Ag_2_O-1 composite nanorods. The primary features include the Ti, Ag, and O peaks that originated from the TiO_2_–AgO composites. The trace carbon contamination on the surface of the nanorod sample originated from exposure to ambient air. No impurity atoms were detected in the nanorod sample. Figure 6b exhibits the Ag 3d core-level doublet spectrum originating from the Ag_2_O decorated via sputtering; two distinct features centered at approximately 367.7 and 373.7 eV respectively correspond to the Ag 3d_5/2_ and Ag 3d_3/2_ binding energies. These binding energies are consistent with the Ag–O binding values reported for the Ag_2_O phase [17]. This indicates that the silver exists in the Ag^+^ valence state in the sputtered Ag_2_O nanoparticles on the composite nanorods studied herein. Figure 6c displays the Ti 2p core-level doublet spectrum associated with the TiO_2_ nanorod template. The distinct two features were deconvoluted into four subpeaks. The subpeaks centered at 458.7 and 464.3 eV correspond to Ti 2p_3/2_ and Ti 2p_1/2_ peaks of the Ti^4+^ valance state, respectively. By contrast, the subpeaks with a relatively weak intensity centered at 457.6 and 463.3 eV correspond to Ti 2p_3/2_ for Ti 2p_1/2_ peaks of the Ti^3+^ valence state [5]. The presence of the mixed Ti^4+^/Ti^3+^ valance state indicates the possible presence of oxygen vacancies in the surfaces of the as-synthesized TiO_2_ nanorods [5,8]. The O1s spectrum of the composite nanorods is shown in Figure 6d. The asymmetric O1s spectrum was deconvoluted into three subpeaks centered at 532.5, 531.2, and 529.2 eV. Notably, the subpeaks centered at 529.2 and 531.2 eV are ascribed to the lattice oxygen in Ag_2_O and TiO_2_, respectively [18,19]. Moreover, the external absorbed −OH groups or water molecules on the surfaces of the composite nanorods are reflected by a subpeak at approximately 532.5 eV [20].

**Figure 7:**
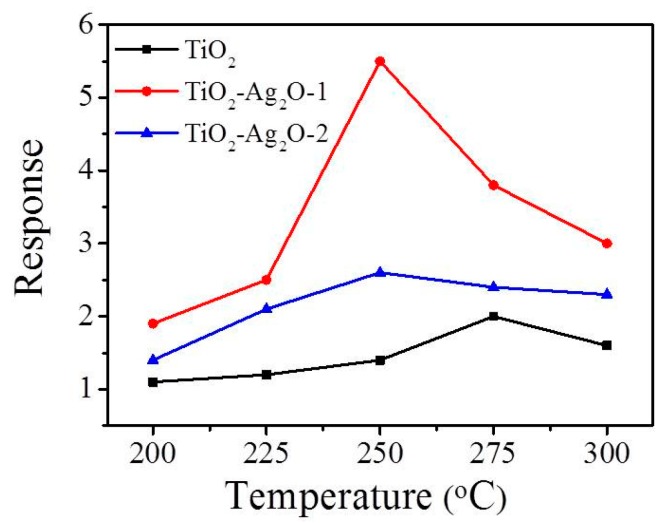
Temperature-dependent gas-sensing responses of various nanorod sensors exposed to 1.5 ppm NO_2_.

Figure 7 shows the temperature-dependent gas-sensing responses to NO_2_ (1.5 ppm) of gas sensors made from TiO_2_, TiO_2_–Ag_2_O-1, and TiO_2_–Ag_2_O-2 composite nanorods. For the NO_2_ target gas, the n-type gas-sensing response of nanorod-based sensors is defined as Rg/Ra and the p-type gas-sensing response of nanorod-based sensors is defined as Ra/Rg, where Rg is the sensor resistance under target gas exposure and Ra is the sensor resistance with the removal of the target gas. The optimal operating temperature of oxide sensors to obtain the highest gas-sensing response is highly associated with the balance between the chemical reactions and the gas diffusion rate of the oxide surfaces [5]. The maximum responses of the TiO_2_–Ag_2_O-1 and TiO_2_–Ag_2_O-2 sensors to NO_2_ were obtained at the operating temperature of 250 °C in this study. Meanwhile, a relatively high operating temperature of 275 °C was needed for the TiO_2_ nanorods to obtain the maximum gas-sensing response under similar gas-sensing test conditions. Notably, the gas-sensing response versus operating temperature curve of TiO_2_–Ag_2_O-1 showed a distinct summit at 250 °C, differing substantially from the curves of the TiO_2_ and TiO_2_–Ag_2_O-2 nanorod sensors. This result might be a sign of different gas-detecting mechanisms operating among the various nanorod-based sensors. Therefore, the optimal gas-sensing temperature of the fabricated composite nanorod sensors toward NO_2_ was chosen as 250 °C in this study.

**Figure 8:**
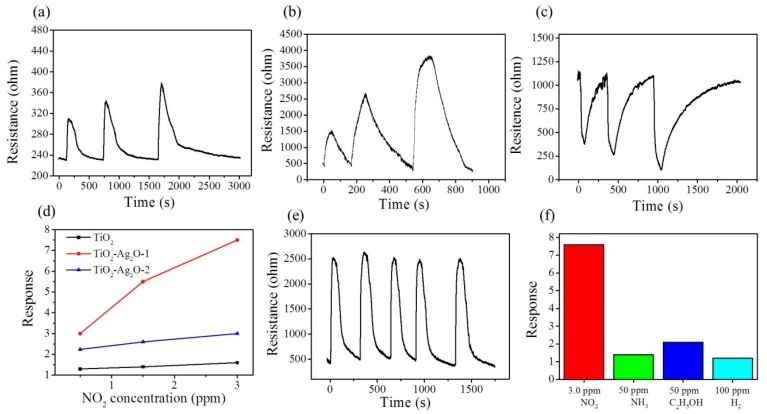
The dynamic response curves of various nanorod sensors to NO_2_ gas ranging from 0.5 ppm to 3.0 ppm: (**a**) TiO_2_, (**b**) TiO_2_–Ag_2_O-1, and (**c**) TiO_2_–Ag_2_O-2. (**d**) Summarized gas-sensing response values versus NO_2_ concentration for various nanorod sensors. (**e**) Cycling gas-sensing tests of TiO_2_–Ag_2_O-1 upon exposure to 1.5 ppm NO_2_ at 250 °C. (**f**) The across selectivity profiles of TiO_2_–Ag_2_O-1 upon exposure to various target gases.

Figure 8a–c shows the dynamic NO_2_ gas-sensing response curves of the TiO_2_, TiO_2_–Ag_2_O-1, and TiO_2_–Ag_2_O-2 sensors, respectively, upon exposure to 0.5–3.0 ppm NO_2_. A sharp increase in sensor resistance was observed for the TiO_2_ and TiO_2_–Ag_2_O-1 nanorod sensors upon exposure to NO_2_; moreover, the sensor resistance decreased with the removal of the NO_2_ target gas (Figure 8a,b). By contrast, the TiO_2_–Ag_2_O-2 showed an opposite sensor resistance variation upon exposure to NO_2_ gas (Figure 8c). This indicates that the TiO_2_ and TiO_2_–Ag_2_O-1 sensors showed an n-type conduction nature and the TiO_2_–Ag_2_O-2 sensor demonstrated a p-type conduction nature during the NO_2_ gas-sensing tests. The aforementioned structural results reveal that the TiO_2_–Ag_2_O-1 sensor exhibited a morphology in which the Ag_2_O particles were dispersedly distributed on the surfaces of the TiO_2_ nanorods. The incomplete coverage of the Ag_2_O particles on the TiO_2_ surfaces of the TiO_2_–Ag_2_O-1 nanorods meant that, upon exposure to the NO_2_ target gas, the n-type conduction dominated the material’s gas-sensing behavior. By contrast, the TiO_2_–Ag_2_O-2 nanorods demonstrated a thick, full-coverage layer of Ag_2_O clusters or aggregations on the surfaces of the TiO_2_ nanorods. This morphology feature might account for the conduction and chemoresistive variation in the TiO_2_–Ag_2_O-2 sensor, which was dominated by p-type Ag_2_O shell layers of the composite nanorods. A similar conduction type variation due to the p-type crystal coverage effect on the p–n heterogeneous oxides has been demonstrated in a ZnO–Cr_2_O_3_ system [11]. Comparatively, the TiO_2_–Ag_2_O-1 sensor exhibited the largest degree of sensor resistance variation before and after the introduction of the NO_2_ gas under the given test conditions. Notably, the pristine TiO_2_ sensor demonstrated the lowest sensor resistance variation size upon expose to NO_2_ gas. The plot of NO_2_ gas-sensing response versus NO_2_ concentration for various TiO_2_ nanorod-based sensors is shown in Figure 8d. The NO_2_ gas-sensing response of the TiO_2_–Ag_2_O-1 sensor was approximately 3.1 upon exposure to 0.5 ppm NO_2_. Moreover, the gas-sensing response of the TiO_2_–Ag_2_O-1 sensor increased to 7.6 upon exposure to 3.0 ppm NO_2_. An approximate increase of the gas-sensing response by 2.4 times was observed with an increase in NO_2_ concentration from 0.5 ppm to 3.0 ppm by the TiO_2_–Ag_2_O-1 sensor. By contrast, the TiO_2_–Ag_2_O-2 sensor exhibited gas-sensing responses of approximately 2.2 and 3.1 upon exposure to 0.5 ppm and 3.0 ppm NO_2_, respectively; these response values are lower than those of the TiO_2_–Ag_2_O-1 sensor under similar test conditions. A concentration-dependent increment of the gas-sensing response for a low concentration range of 0.5–3.0 ppm NO_2_ was less visible for the TiO_2_–Ag_2_O-2 sensor. Notably, the gas-sensing response of the pristine TiO_2_ sensor at the same operating temperature did not show a response value larger than 2.0, revealing that the decoration of discrete or layered Ag_2_O particles or aggregations on the surfaces of TiO_2_ nanorods to form a p–n heterogeneous system is beneficial to the enhancement of the NO_2_ gas-sensing response of TiO_2_ nanorods. The gas-sensing response time of the nanorod-based sensors is defined as the duration required for an occurrence of a 90% change in sensor resistance upon exposure to the target gas, while the recovery time is the duration in which the sensor resistance drops by 90% from the maximal steady-state value, following the removal of the target gas. The response times for the TiO_2_, TiO_2_–Ag_2_O-1, and TiO_2_–Ag_2_O-2 nanorod sensors upon exposure to 0.5–3.0 ppm NO_2_ gas ranged from 85 to 93 s. No substantial difference in the response times of various nanorod sensors exposed to different concentrations of NO_2_ gas was observed. By contrast, a marked improvement in the recovery time of the TiO_2_ nanorods sputtered with a coating of Ag_2_O particles was visibly demonstrated. The recovery times of the pristine TiO_2_ nanorod sensor ranged from 405 to 820 s after exposure to 0.5 to 3.0 ppm NO_2_. Decreased recovery times were shown in the TiO_2_–Ag_2_O-2 nanorod sensor, which ranged from 191 to 280 s after exposure to 0.5 to 3.0 ppm NO_2_. Notably, the TiO_2_–Ag_2_O-1 nanorod sensor exhibited a substantial decrease in the recovery time upon the removal of NO_2_ gas; the recovery times ranged from 97 to 136 s in the NO_2_ concentration range of 0.5 to 3.0 ppm. The size of the Ag_2_O particles (or clusters) and their dispersibility are vital factors affecting gas-sensing performance, which lead to the highly effective desorption of surface-adsorbed ions with the removal of the target gas at elevated temperatures [21]. The TiO_2_–Ag_2_O-1 nanorod sensor exhibited the superior gas-sensing performance among the various nanorod sensors in this study. The cycling gas-sensing tests of the TiO_2_–Ag_2_O-1 nanorod sensor exposed to 1.5 ppm NO_2_ at 250 °C are shown in Figure 8e. The result indicates that the TiO_2_–Ag_2_O-1 nanorod sensor had good reproducibility during multiple cycles of response and recovery. Figure 8f shows the across selectivity profiles of the TiO_2_–Ag_2_O-1 sensor upon exposure to 100 ppm H_2_, 50 ppm C_2_H_5_OH, and 50 ppm NH_3_ gases, as well as 3.0 ppm NO_2_. The TiO_2_–Ag_2_O-1 sensor exhibited a highly selective gas-sensing response toward the low-concentration NO_2_ gas as compared to the other various target gases. 

The NO_2_ gas-sensing performances of the sensors based on several TiO_2_-based composite oxides are summarized in Table 1. Compared to previous works [22,23,24,25], the TiO_2_–Ag_2_O-1 nanorod sensor herein showed superior NO_2_ gas-sensing performance under similar test conditions. The gas-sensing test results herein demonstrated that the TiO_2_–Ag_2_O composite nanorods decorated with discrete Ag_2_O particles have potential for applications as NO_2_ gas sensors at low concentrations. The possible surface chemisorption reactions occurring during the gas-sensing process of the TiO_2_–Ag_2_O composite nanorods upon exposure to NO_2_ gas are described below:

(1) $${\mathrm{NO}}_{2}+e^{-}\to {\mathrm{NO}}_{2}^{-},$$

(2) $${\mathrm{NO}}_{2}+O_{2}^{-}+2e^{-}\to {\mathrm{NO}}_{2}^{-}+2O^{-}.$$

**Figure 9:**
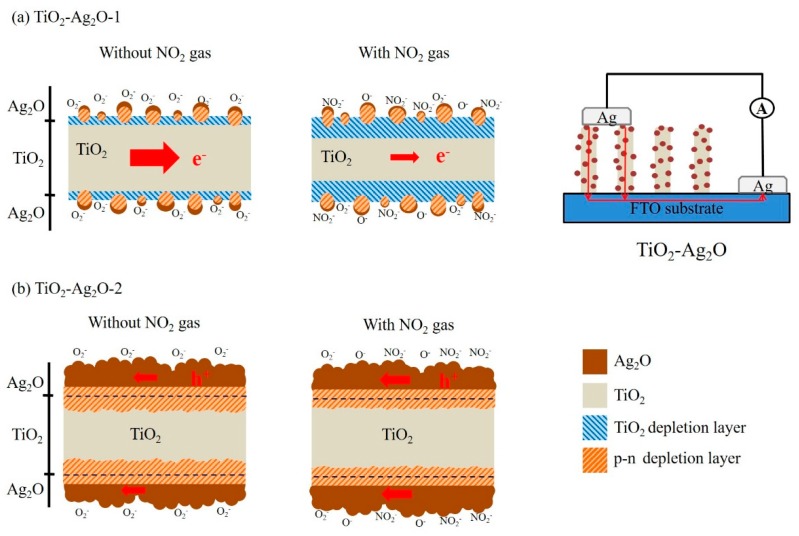
Schematic illustrations for possible gas-sensing mechanisms of (**a**) TiO_2_–Ag_2_O-1 and (**b**) TiO_2_–Ag_2_O-2 toward NO_2_ gas.

The NO_2_ molecules capture electrons from the oxide surface to form NO_2_^−^ ions; this engenders the electron density variation of the oxides. By contrast, the surface-adsorbed NO_2_^−^ ions are desorbed with the removal of the NO_2_ gas and, consequently, in this process the recovery of the initial conditions takes place. Notably, the contact of the TiO_2_–Ag_2_O oxides form p–n junctions at the hetero-interfacial regions. This additionally formed potential barrier in the TiO_2_–Ag_2_O composite nanorods explains the superior gas-sensing responses of the composite nanorods compared to that of the pristine TiO_2_ nanorods. A similar formation of heterogeneous p–n junctions improves the gas-sensing responses of composite nanorods, as has been demonstrated in ZnO–ZnCr_2_O_4_, ZnO–Mn_3_O_4_, and ZnO–Cr_2_O_3_ p–n composite structures [9,10,11]. Furthermore, the reasons for the NO_2_ gas-sensing response of the TiO_2_–Ag_2_O-1 sensor being higher than that of the TiO_2_–Ag_2_O-2 sensor at the given test conditions are explained by the schematic mechanisms exhibited in Figure 9. The schematic of the gas sensor device is also shown in Figure 9a. When the Ag_2_O particles are coated on the surfaces of the TiO_2_ nanorods in a discrete configuration, the randomly distribution of the depletion region at the interface of the p-Ag_2_O and n-type TiO_2_ will initially narrow the space of the conducting channel along the radial direction of the TiO_2_ (Figure 9a). Moreover, the exposure of the free surfaces of the Ag_2_O particles and TiO_2_ rods in ambient air also initially lead to a surface hole accumulation layer and depletion layer, respectively. Furthermore, following the decoration of the Ag_2_O particles in a continuous layer configuration on the surfaces of the TiO_2_ nanorods, the conducting channel in the TiO_2_ will also be narrowed (Figure 9b). After introducing NO_2_ gas into the test chamber, the depletion region size at the TiO_2_–Ag_2_O hetero-interfacial region of the TiO_2_–Ag_2_O-1 sensor varies due to the surface-adsorbed NO_2_^⁻^ ions. Moreover, the surface depletion region of the TiO_2_ nanorods is also thickened. The variation of the depletion size at different regions, further narrowing the conduction channel size of the TiO_2_ nanorods, results in the increased sensor resistance of the TiO_2_–Ag_2_O-1 nanorod sensor. By contrast, the TiO_2_–Ag_2_O-2 nanorod sensor exhibits a p-type conduction gas-sensing behavior in this study. This reveals that the conduction channel size in the TiO_2_ nanorod of the composite nanorod no longer plays a vital role affecting the chemoresistive variation upon exposure to NO_2_ gas. It has also been shown that in core–shell ZnO–ZnMn_2_O_4_ and ZnO–Cr_2_O_3_ nanostructures, the p–n contact regions at the hetero-interfaces no longer play significant roles in the gas-sensing reaction [10,11]. The conduction path in the Ag_2_O layer, by contrast, dominates the gas-sensing response of the TiO_2_–Ag_2_O-2 nanorod sensor. Notably, when the TiO_2_–Ag_2_O-2 nanorod is exposed to NO_2_ gas, the accumulation layer in the Ag_2_O layer thickens. This increases the carrier number in the p-type Ag_2_O layer; therefore, a decreased sensor resistance is expected. However, the surface Ag_2_O layer-dominated chemoresistive variation size of the TiO_2_–Ag_2_O-2 nanorod sensor is expected to be lower than that of the TiO_2_–Ag_2_O-1 nanorod sensor, which is dominated by the rugged conduction channel size in the TiO_2_ core region upon exposure to NO_2_ gas. Therefore, the superior NO_2_ gas-sensing performance was obtained by the TiO_2_–Ag_2_O-1 nanorod sensor in this study.

## 4. Conclusions

In summary, TiO_2_–Ag_2_O composite nanorods were synthesized through the combination of hydrothermal growth and sputtering methods. The structural analysis reveals that the as-synthesized TiO_2_–Ag_2_O composite nanorods have a high crystallinity. The electron microscopy analysis results demonstrate that a shorter Ag_2_O sputtering duration causes the formation of TiO_2_–Ag_2_O composite nanorods decorated with discrete Ag_2_O particles. Meanwhile, a prolonged Ag_2_O sputtering process engenders the aggregation of numerous Ag_2_O particles, which form a layered configuration on the composite nanorods. The formation of p–n junctions in the composite nanorods enhances their NO_2_ gas-sensing performance as compared to pristine TiO_2_ nanorods. Moreover, different gas-sensing mechanisms of the TiO_2_–Ag_2_O nanorods with various Ag_2_O coverage morphologies account for the superior NO_2_ gas-sensing responses of the TiO_2_–Ag_2_O-1 sensor at a low concentration range in this study.

## Figures and Tables

**Table 1:** NO_2_ gas-sensing performance of various TiO_2_-based composites prepared using various methods in the operating temperature range of 200–300 °C [22,23,24,25].

Composite Nanorods	Synthesis Method	Operating Temperature (°C)	Concentration (ppm)	Response (Ra/Rg)	Detection Limit (ppm)	Response/Recovery Time (s)
TiO_2_–Ag_2_O (this work)	Hydrothermal and sputtering method	250	1.5	5.5	0.5	87/112
TiO_2_–Er_2_O_3_	Sol-gel method	200	10	4.5	0.5	N/A
TiO_2_–V_2_O_5_	Sol–gel and solvothermal method	200	2	0.8	N/A	N/A
TiO_2_–MoO_3_	Sol–gel method	300	2	2.3	0.5	120/180
TiO_2_–Ga_2_O_3_	Sol–gel method	200	2	2.25	N/A	150/270
